# Supplementary material for: Improved Survival after Transarterial Radioembolisation for Hepatocellular Carcinoma Gives the Procedure Added Value
Source: J Clin Med. 2022 Dec 16;11(24):7469. doi: 10.3390/jcm11247469 (PMC9781303; doi:10.3390/jcm11247469)
Supplement: Supplementary file 1 [file jcm-11-07469-s001.zip › jcm-2013061-supplementary.pdf]

Supplementary material

S1. Detailed selection criteria

The main selection criteria were: (a) age ≥18 years; (b) perceived life expectancy ≥3 months; (c) Eastern Cooperative Oncology Group (ECOG) Performance Status (PS) ≤1 and (d) serum bilirubin ≤2 mg/dl. The main exclusion criteria were: (a) tumour extent exceeding 50% of the whole liver volume; (b) non-measurable tumour burden; (c) extrahepatic metastases; (d) Child - Pugh score> 8; (f) serum creatinine> 2 mg/dl; (g) pulmonary insufficiency; (h) previous radiotherapy (i) previous transplant or other immunodeficient condition; (j) contraindication for angiography or selective visceral catheterisation; (k) evidence of hepatopulmonary shunt> 20% on scintigraphy with technetium-99 m macroaggregated albumin (99mTc-MAA) and (m) evidence of any detectable release of 99mTc-MAA into the stomach or duodenum after gastroduodenal artery embolisation (if necessary).

S2. Adverse events definition

Imaging and laboratory tests were repeated at 1, 3 and 6 months and were subsequently scheduled by the referring physician. Adverse events (AEs) were prospectively recorded and herein redefined according to the National Cancer Institute's Common Terminology Criteria Adverse Events (NCI-CTCAE) version 3.0 [20]. Radiation-induced liver disease (RILD) was always graded as 3-4 and defined as the appearance, early after TARE, of at least one of the following: ascites, variceal haemorrhage, hepatic encephalopathy, total bilirubin> 3 mg/dL and an International Normalised Ratio (INR)>2.0. Ascites was classified as grade 1-2 when it did not require invasive surgery and as grade 3-4 when it required drainage or resulted in life-threatening consequences. Pulmonary events included radiation pneumonia and were classified as grade 1-2 when they were asymptomatic or required medical intervention only, and as grade 3-4 when severe symptoms occurred, requiring oxygen or even urgent intervention. Acute cholecystitis was classified as grade 1-2 when invasive interventions were not required and as grade 3-4 when invasive interventions were required or resulted in life-threatening consequences. Fever was graded as 1-2 when it was < 40°C and as 3-4 when > 40°C with clinical and laboratory signs of sepsis. Fatigue was graded as 1-2 when relieved by rest or by limiting personal care activities of daily living; otherwise ,it was graded as 3. Finally, gastrointestinal events included perforation and haemorrhage, and were classified as 3-4 when requiring endoscopic or surgical interventions.
